# Bridging the Digital Divide: Factors Influencing eHealth Use Among Homebound Older Adults During the COVID-19 Pandemic

**DOI:** 10.1177/07334648251343407

**Published:** 2025-05-15

**Authors:** Joonhyeog Park, BoRin Kim, Joonyoung Cho, Sojung Park

**Affiliations:** 1 6572 School of Social Policy & Practice, University of Pennsylvania, Philadelphia, PA, USA; 2 3067 College of Health and Human Services, University of New Hampshire, Durham, NH, USA; 3 3949 Thompson School of Social Work and Public Health, University of Hawai'i at Mānoa, Honolulu, HI, USA; 4 Brown School, Washington University in St. Louis, St. Louis, MO, USA

**Keywords:** COVID-19, information technology, health services, homebound older adults, digital divide

## Abstract

This study examined how access to Information and Communication Technology (ICT) devices, prior online experience, and ICT training (with or without assistance) influenced eHealth use among homebound Medicare beneficiaries in the U.S. during COVID-19. Data were obtained from the National Health and Aging Trends Study, and participants (*N* = 653) were categorized as non-users, patient portal users, video telehealth users, or dual users. Multinomial logistic regression models showed that access to ICT devices was initially associated with eHealth engagement. However, this association became non-significant after accounting for prior online experience and ICT training. Prior online experience significantly predicted patient portal use, while ICT training, particularly when provided with assistance, significantly predicted video telehealth use and dual usage. The study highlights that providing ICT devices alone may be insufficient to reduce eHealth disparities among homebound older adults. Educational programs promoting digital engagement and targeted training are essential to ensure equitable healthcare access.

**What this paper adds**

• An analysis of eHealth engagement rates among U.S. homebound older adults during the early stages of the COVID-19 pandemic, categorizing them as non-users, patient portal users, video telehealth users, or dual users.
• An examination of the relative influence of access to ICT devices, prior online experience, and ICT training (with and without assistance) on eHealth adoption.
• Policy and practice recommendations to improve eHealth engagement among homebound older adults.

**Applications of study findings**

• While providing ICT devices is crucial in promoting eHealth engagement, this study demonstrates that material access alone is insufficient. Experience with digital tools and ICT training is essential to bridging eHealth adoption gaps among homebound older adults.
• Tailored eHealth engagement strategies should consider specific technology needs, with prior online experience driving patient portal use and ICT training, with assistance facilitating video telehealth and dual usage.
• Findings highlight the limitations of self-directed ICT training modules, emphasizing the importance of in-person assistance for device setup and ongoing support to ensure successful eHealth adoption.

## Introduction

Advances in information and communication technologies (ICTs) in healthcare are rapidly transforming patient experiences and access to care. Among these advances, *eHealth* stands out by leveraging digital technologies to enhance healthcare delivery. eHealth encompasses a broad spectrum of applications, with notable examples such as patient portals—secure platforms for messaging and online medical record management—and video telehealth services, which facilitate real-time medical consultations via video calls (Nahm et al., 2020; Shaw et al., 2017; Vergouw et al., 2020; Wilson et al., 2021).

eHealth has proven particularly beneficial for homebound older adults who are unable to leave their homes or require assistance due to multiple chronic conditions and significant functional limitations. In the U.S., an estimated 2 million older adults rarely or never leave their homes, while more than 5 million can do so only with difficulty or assistance (Ankuda et al., 2022; Ornstein et al., 2015). These individuals face substantial barriers in accessing traditional in-person healthcare services, including mobility limitations, transportation challenges, and a lack of caregivers to assist with appointments (Federman, 2021; Franzosa, 2023; Harrison, 2022). eHealth helps bridge this gap by enabling timely, personalized care through internet-connected devices and platforms, reducing travel costs and minimizing exposure to infectious diseases such as COVID-19 and influenza (Goldberg, 2021; Kalicki, 2021). eHealth use among homebound older adults has been linked to improved overall health, medication adherence, social interaction, and reduced depression and disability (Choi et al., 2014, 2020; Fjellså et al., 2022; Gellis et al., 2012; Wong et al., 2021).

Despite these benefits, eHealth use among older adults remains limited due to a persistent digital divide, especially for those unfamiliar with technology or lacking devices (Choi et al., 2024; Falvey et al., 2024; Nahm et al., 2020; Wilson et al., 2021). Approximately 41% of Medicare beneficiaries lack access to the high-speed internet or smartphones required for eHealth, and 38% are unprepared for video telehealth due to technological inexperience (Lam et al., 2020; Roberts & Mehrotra, 2020). These disparities persisted during the COVID-19 pandemic, with adults aged 50 and older continuing to experience more difficulties with online visits (50.3% vs. 28.6%) than younger adults, even as overall use increased after the outbreak (Pasquinelli et al., 2022). Homebound older adults, who frequently experience financial strain, functional limitations, and chronic conditions, face even more pronounced digital inequities (Kalicki et al., 2021; Loizos et al., 2023). For example, a seven-year longitudinal study found that older adults with serious illnesses—71% of whom were homebound—had significantly lower ICT access and proficiency than the broader older adult population (Frydman et al., 2021).

While efforts to provide ICT devices, promote usage experience, and offer training are vital, their effectiveness among homebound older adults remains uncertain. Studies during COVID-19 have linked access to digital tools, prior online experience, and support or training to increased eHealth use among older adults (Choi et al., 2022; Chung et al., 2021; Kim & Ang, 2023; Ng et al., 2022; Qin, 2022). However, these studies have not explicitly focused on homebound individuals, whose unique challenges may limit intervention effectiveness. Moreover, little is known about how access, experience, and training relate to different eHealth modalities, such as patient portals, video telehealth, or both. Even when devices are available, unfamiliarity with technology often hinders engagement (Kalicki et al., 2021; Loizos et al., 2023), suggesting that material access alone may be insufficient. A more nuanced understanding of these factors is essential to address eHealth disparities in this population.

### Present Study

This study investigates how access to ICT devices, prior online experience, and ICT training (with or without assistance) are associated with eHealth use among homebound older adults during the COVID-19 pandemic. Using a nationally representative sample of Medicare beneficiaries, we address the following research question and hypothesis:

#### Research Question

To what extent are access to ICT devices, prior online experience, and ICT training (with and without assistance) associated with eHealth use among homebound older adults during the COVID-19 pandemic?

Hypothesis We hypothesize that access to ICT devices will be positively associated with eHealth use among homebound older adults. However, this association will diminish when prior online experience and ICT training are included in the model. Prior online experience and ICT training (both with and without assistance) are expected to show stronger positive associations with eHealth use.

## Methods

### Data and Sample

This study used public-use data files from the National Health and Aging Trends Study (NHATS) main surveys for 2019–2020, supplemented with the 2020 COVID-19 survey. NHATS is a longitudinal study that annually collects data from a nationally representative panel of Medicare beneficiaries aged 65 and older. Due to the pandemic, the 2020 main survey (Round 10) was conducted via telephone. Participants who completed the 2020 main survey were subsequently administered a supplementary COVID-19 survey from June 2020 through January 2021 to gather data on their pandemic experiences. The 2020 main survey included 3961 participants, 3257 of whom provided complete data for the COVID-19 survey.

We restricted our analytic sample to homebound older adults aged 70 and older, following NHATS guidelines to ensure consistency in population-based analyses (NHATS, 2022). This age restriction enhances comparability with prior NHATS research and improves representativeness across survey rounds. Furthermore, focusing on individuals aged 70 and above aligns with our research objective of examining eHealth use among homebound older adults. This group tends to be older, experiences more significant functional limitations, and faces greater barriers to digital access and use (Choi et al., 2024).

Homebound status was determined based on responses to the 2020 NHATS main survey question: “In the last month, how often did you leave your building/home to go outside?” Response options ranged from every day (7 days a week) to never. In line with prior studies, we classified participants as homebound if they reported leaving home rarely (once a week or less) or never. Additionally, participants were considered homebound if they left home some days but reported substantial difficulty doing so, rarely or never went out independently, or were unable to leave due to lack of assistance (Ankuda et al., 2022; Choi et al., 2024; Ornstein et al., 2015). The final analytic sample included 653 participants, 83% of whom reported rarely or never leaving home in the past month.

### Measures

#### Dependent Variable

***eHealth use*** was measured using a question from the COVID-19 survey: “During the COVID-19 outbreak, how did you communicate with your usual healthcare provider?” Participants who answered “yes” to both “emails, texts, or portal messages” and “video telehealth” were categorized as dual users. Those who used only video telehealth were labeled video telehealth users, and those who used only emails, texts, or portal messages were labeled patient portal users. Those who answered “no” to both were classified as non-users.

#### Key Independent Variables

***Access to ICT devices*** was measured using three questions from the 2020 main survey: (1) “Do you have a working cell phone?” (2) “Do you have a tablet computer like the iPad that works by touching the screen?” and (3) “Do you have a working computer in your home, apartment, room, unit, suite, or other living space?” Participants who answered “yes” to any of these questions were considered to have ICT access. To account for potential differences in how these devices support eHealth engagement, we categorized access to ICT devices into two separate variables: Cell phone access and computer/tablet access (1 = yes; 0 = no). This distinction is important because computers/tablets are better suited for tasks involving larger screens and complex navigation, whereas cell phones primarily facilitate communication. Additionally, since the survey did not specify whether reported cell phones were smartphones, we analyzed cell phone access separately to minimize misclassification bias.

***Prior online experience*** was assessed with one question from the 2020 main survey: “In the last month, have you ever gone on the Internet or online for any reason?” Responding “yes” to this question was coded dichotomously (1 = yes, indicating prior online experience; 0 = no).

***ICT Training*** was assessed with two questions from the COVID-19 survey: (1) “During the COVID-19 outbreak, have you learned a new technology or program to go online? This includes learning to use a smartphone, computer, or iPad, or a program like Zoom or FaceTime.” (2) “Has anyone helped you with that, or did you learn that on your own?” Based on the responses to these two consecutive questions, an ICT training variable was created with three categories: 1 = no; 2 = yes, but without assistance; 3 = yes, with assistance.

#### Covariates

The study included several covariates that may influence eHealth use among homebound older adults (Choi et al., 2022; Kim & Ang, 2023; Qin, 2022). Sociodemographic characteristics include 1) age group (1 = 80+; 0 = 70s), (2) gender (1 = female; 0 = male), (3) race/ethnicity (1 = non-White [including all racial/ethnic groups except non-Hispanic White]; 0 = non-Hispanic White), (4) metropolitan area residency (1 = metro residency; 0 = non-metro residency), and (5) marital status (1 = married/partnered; 0 = not married/partnered). As household income was not assessed in the 2020 main survey, we used a log-transformed, imputed income variable from the 2019 main survey. Additionally, we controlled for several health condition variables: (1) the number of activities of daily living (ADLs) limitations for which participants needed assistance during COVID-19 (eating, showering/bathing, dressing, toileting, getting out of bed, and assistance inside the home), (2) the number of diagnosed chronic medical conditions (heart attack, heart disease, hypertension, stroke, arthritis, osteoporosis, diabetes, lung disease, dementia/Alzheimer, or cancer), and (3) living in a facility during COVID-19, including independent living, nursing homes, or other residential settings that assist with daily activities (1 = yes; 0 = no, i.e., living in the community).

### Analytic Strategies

The statistical analysis was conducted in two main steps. First, we used chi-square tests and one-way ANOVA to examine sample characteristics across the four eHealth use categories. Second, we conducted hierarchical multinomial logistic regression to assess the associations between ICT-related factors and eHealth use. Model 1 included access to ICT devices along with sociodemographic and health-related covariates. Model 2 added prior online experience, and Model 3 included ICT training (with and without assistance). Given the relatively small group sizes, we used robust standard errors to improve the reliability of estimates. We also conducted Wald tests to assess whether each ICT-related variable significantly improved model fit (Fox, 1997). All analyses were performed using Stata 18.

## Results

### Sample Characteristics by Types of eHealth Use

Table 1 presents the characteristics of the overall sample, categorized into non-users, patient portal users, video telehealth users, and dual users. Across all groups, there were no significant differences in age, gender, metropolitan residency, marital status, or the number of chronic medical conditions. The sample primarily consisted of individuals aged 80 or older (71.7%), the majority were female (73.5%), and most lived in metropolitan areas (83.8%). Additionally, 30.9% of participants were married or in a partnership, and the average number of chronic medical conditions was 3.5.

**Table 1.** Sample Characteristics by Types of eHealth Use.

	Total	Non-users	Patient portal users	Video telehealth users	Dual users	*p*
	*N* = 653 (100%)	*N* = 477 (73.0%)	*N* = 39 (6.0%)	*N* = 97 (14.9%)	*N* = 40 (6.1%)	
Age group (%)						.141
70s	28.3	26.0	38.5	35.1	30.0	
80+	71.7	74.0	61.5	64.9	70.0	
Female (%)	73.5	75.1	56.4	71.1	77.5	.070
Non-White (%)	39.4	37.5	28.2	48.5	50.0	.047
Metro residency (%)	83.8	82.8	87.2	87.6	82.5	.622
Married/partnered (%)	30.9	28.9	38.5	37.1	32.5	.295
Log HH income (M, SE)	10.2 (1.2)	10.1 (1.3)	10.6 (0.8)	10.1 (1.3)	10.5 (0.8)	.035
ADL limitations (M, SE)	1.7 (2.3)	1.7 (2.2)	0.8 (1.8)	2.3 (2.5)	2.1 (2.2)	.004
Chronic conditions (M, SE)	3.5 (1.5)	3.5 (1.4)	3.4 (1.7)	3.7 (1.5)	3.9 (1.5)	.325
In assisted living facility (%)	21.3	22.0	7.7	27.8	10.0	.019
ICT access—cell phone (%)	65.4	61.8	89.7	70.1	72.5	.002
ICT access—PC/tablet (%)	57.0	52.2	87.2	59.8	77.5	<.001
Prior online experience (%)	29.7	23.5	76.9	34.0	47.5	<.001
ICT training (%)						<.001
No training	81.7	86.8	76.3	67.4	59.3	
Without assistance	5.9	4.6	10.5	9.0	9.4	
With assistance	12.4	8.6	13.2	23.6	31.3	

The largest group in the sample was non-users, comprising 73.0% of participants. Within this group, 37.5% identified as non-White and had relatively lower household incomes. On average, they required assistance with 1.7 ADLs, similar to the overall sample. This group was also more likely to live in institutionalized settings (22.0%). Engagement with ICT was relatively low, with 61.8% owning a cell phone and 52.2% having a computer or tablet at home. Only 23.5% reported prior online experience and 13.2% received ICT training during the pandemic, with 8.6% learning with assistance.

Patient portal users accounted for 6.0% of the sample and differed notably from non-users. This group had the lowest percentage of non-White individuals (28.2%), higher household incomes, and a lower likelihood of institutional residence (7.7%). They also had significantly fewer ADL limitations (0.8). ICT engagement was high, with 89.7% owning a cell phone and 87.2% having a computer or tablet. Additionally, 76.9% had prior online experience and 23.7% received ICT training, including 13.2% who learned with assistance.

Video telehealth users represented 14.9% of the sample and had household incomes comparable to non-users. This group had a relatively higher percentage of non-White individuals (48.5%) and the highest level of ADL dependency, requiring assistance with an average of 2.3 ADLs. They were also more likely than non-users to reside in institutional settings (27.8%). ICT engagement was moderate, with 70.1% owning a cell phone, and 59.8% having a computer or tablet. In addition, 34.0% had prior online experience and 32.6% received ICT training, with 23.6% learning with assistance.

Dual users made up 6.1% of the sample and showed the most significant differences from non-users. This group had the highest proportion of non-White participants (50.0%), higher household incomes, and lower rates of institutional residence (10.0%). On average, dual users reported 2.1 ADL limitations. ICT engagement was relatively high, with 72.5% owning a cell phone and 77.5% having a computer or tablet. Nearly half (47.5%) had prior online experience and 40.7% received ICT training, including 31.3% who learned with assistance.

### Associations Between eHealth Use, Access to ICT Devices, Prior Online Experience, and ICT Training

Table 2 presents results from multinomial logistic regression analyses, with three sequential models examining the associations between eHealth use type and ICT access, prior online experience, and ICT training, while adjusting for covariates. In Model 1, which included access to ICT devices and covariates, having a cell phone or computer/tablet was significantly associated with an increased likelihood of eHealth engagement. Specifically, cell phone access was significantly associated with higher relative risk of being a patient portal user (RRR = 3.167, *p* < .05) and marginally associated with video telehealth use (RRR = 1.820, *p* < .10). Similarly, access to a computer/tablet was significantly associated with patient portal use (RRR = 3.146, *p* < .05) and dual use (RRR = 2.501, *p* < .05), with a marginal association with video telehealth use (RRR = 1.658, *p* < .10).

**Table 2.** Association Between eHealth Use, Access to ICT Devices, Prior Online Experience, and ICT Training.

	Model 1: RRR^b^			Model 2: RRR^b^			Model 3: RRR^b^		
	Patient portal users^a^	Video telehealth users^a^	Dual users^a^	Patient portal users^a^	Video telehealth users^a^	Dual users^a^	Patient portal users^a^	Video telehealth users^a^	Dual users^a^
Age—80+	0.750	0.680	0.966^**+**^	1.015	0.753	1.184	1.189	0.808	1.040
Female	0.422*	0.938	1.309	0.359**	0.894	1.184	0.382*	0.799	1.328
Non-White	0.697	1.835*	2.378*	0.879	1.958**	2.705**	0.975	2.084**	2.167^**+**^
Metro residency	1.026	1.441	0.749	0.887	1.371	0.701	0.816	1.325	0.941
Married/partnered	0.495^**+**^	1.345	0.601	0.455^**+**^	1.277	0.569	0.533	1.222	0.695
Log household income	1.465	0.991	2.104**	1.230	0.972	1.913**	1.237	0.980	1.821*
ADL limitations	0.943	1.198**	1.207*	1.064	1.224**	1.282**	1.034	1.255**	1.295**
Chronic conditions	1.073	1.058	1.197	1.081	1.067	1.205	1.119	1.100	1.168
In assisted living facility	0.657	2.088*	0.495	0.570	1.958*	0.428	0.563	2.076^**+**^	0.344
ICT access—cell phone	3.167*	1.820^**+**^	1.284	1.828	1.630	0.995	1.780	1.747	1.029
ICT access—PC/tablet	3.146*	1.658^**+**^	2.501*	1.350	1.348	1.753	1.309	1.368	1.217
Prior online experience				8.082***	1.872^**+**^	3.295*	8.404***	1.284	2.006
ICT training									
Without assistance							0.900	2.301	1.948
With assistance							0.597	3.619***	4.090**
Model fit improvement: Wald test	19.59**			21.62***			20.83**		

^a^RRR = relative risk ratio.

^b^Reference is non-users.

+*p* < .10 *p < .05, **p < .01, and ***p < .001

In Model 2, which added prior online experience, the associations between ICT device access and eHealth use were no longer statistically significant. In contrast, prior online experience was significantly associated with a greater likelihood of being a patient portal user (RRR = 8.082, *p* < .001), marginally associated with video telehealth use (RRR = 1.872, *p* < .10), and significantly associated with dual use (RRR = 3.295, *p* < .05).

In Model 3, which added ICT training (with and without assistance), prior online experience remained the strongest predictor of patient portal use (RRR = 8.404, *p* < .001). Additionally, receiving ICT training with assistance was significantly associated with a higher relative risk of being a video telehealth user (RRR = 3.619, *p* < .001) and a dual user (RRR = 4.090, *p* < .01). However, ICT training without assistance was not significantly associated with any form of eHealth engagement.

Wald tests were conducted to evaluate the contribution of each ICT-related variable to model fit. In Model 1, access to cell phones or computers/tablets significantly improved model fit (χ^2^ = 19.59, *p* < .01). This was followed by an even greater improvement from prior online experience in Model 2 (χ^2^ = 21.62, *p* < .001) and further improvement with ICT training in Model 3 (χ^2^ = 20.83, *p* < .01). These findings underscore that, while all three variables contribute significantly to explaining eHealth use, prior online experience and ICT training (particularly with assistance) have stronger predictive power than simple access to ICT devices.

## Discussion

The shift toward digital healthcare presents both opportunities and challenges for homebound older adults requiring continuous and secure medical care. However, little was previously known about this group’s utilization of eHealth or the role of access to ICT devices, prior online experience, and ICT training in promoting engagement, particularly during the COVID-19 pandemic. This study addressed that gap using nationally representative data from U.S. Medicare beneficiaries. Participants were categorized as non-users, patient portal users, video telehealth users, or dual users, based on their engagement with these services. We compared characteristics across these groups and examined the relative influence of ICT-related factors on eHealth use.

Findings revealed that nearly three-quarters of the sample did not use eHealth, underscoring a substantial gap in digital health adoption among homebound older adults. Among eHealth users, video telehealth was the most common modality during the pandemic (14.9%), followed by dual users (6.1%) and patient portal users (6.0%). Compared to prior research not restricted to homebound adults, which reported 23.6% using patient portals and 21.1% using video telehealth (Choi et al., 2022), our findings suggest that homebound older adults have lower utilization of patient portals but a comparable reliance on video telehealth. Furthermore, the higher proportion of individuals using only one service rather than both suggests that engagement with one type of eHealth does not necessarily facilitate engagement with another.

Group differences in demographic and health characteristics were also evident. Non-users and video telehealth users were more likely to have lower incomes and to reside in care facilities, whereas patient portal and dual users were generally from higher-income households and were less likely to be institutionalized. Video telehealth and dual users reported the greatest need for assistance with ADLs, followed by non-users and patient portal users. Notably, nearly half of the video telehealth and dual users identified as non-White, compared to a lower representation among non-users and patient portal users.

Multivariate analyses revealed two key findings with significant policy and practice implications. First, the relative influence of ICT-related variables differed in meaningful ways. While access to cell phones or computers/tablets was initially associated with increased eHealth use, these associations diminished after including prior online experience and ICT training in the models. Ultimately, only prior online experience and ICT training with assistance remained significant predictors. This finding echoes prior studies showing that device provision alone is insufficient for meaningful engagement. For example, Loizos et al. (2023) reported that only one-third of homebound patients could use eHealth services after receiving devices, primarily due to limited digital literacy. Similarly, Finkelstein et al. (2023) found that older adults with prior tech experience were better able to benefit from support services, as they could identify their needs and seek help accordingly. Haase et al. (2021) further emphasized the importance of technological assistance from close relationships, noting that older adults who received immediate support were more likely to engage in online activities, as such assistance helped them overcome usage barriers. Taken together, these findings highlight the need for sustained digital engagement efforts and skills training, not just device distribution, to promote eHealth adoption.

Although recent federal initiatives (e.g., the Affordable Connectivity Program [2021], Broadband Equity, Access, and Deployment Program [2022], and Digital Equity Act [2021]) aim to improve internet access among marginalized populations, discussions around skills training and support remain limited (Sheon & Khoong, 2024). Expanding funding and policy support for educational programs could help bridge the digital divide more effectively. Additionally, smaller clinics, rural hospitals, and safety-net providers often face infrastructural, financial, or technological constraints in offering eHealth services (Goldberg, 2021; Hirko et al., 2020; Kalicki et al., 2021). To ensure equitable eHealth access, provider-side implementation must also be strengthened through investment in infrastructure, training, and support mechanisms.

Our findings also demonstrate that different modalities of eHealth use are influenced by different factors. In the final model, prior online experience was the strongest predictor of patient portal use, whereas ICT training with assistance was most predictive of video telehealth and dual use. These results diverge slightly from prior studies that showed strong associations between both predictors and both modalities (Choi et al., 2022; Kim & Ang, 2023; Qin, 2022). One possible explanation is our approach to categorizing users into mutually exclusive groups, which allowed us to better differentiate use patterns across services. Patient portals are typically used for viewing health records or scheduling appointments (Anthony et al., 2023), and their use aligns with familiarity with web-based tools such as email, online banking, or shopping. By contrast, video telehealth requires real-time interaction and technical troubleshooting, which may explain the stronger association with assisted training (Kalicki et al., 2021; Loizos et al., 2023).

These findings support the need for tailored strategies to engage different eHealth user types. For instance, helping users create email accounts could remove a common barrier to patient portal registration (Kruse et al., 2020). Likewise, designing portal interfaces to resemble familiar platforms like Facebook or WhatsApp could improve usability, supported by evidence linking social media use to greater portal engagement (Otokiti et al., 2020). Because assisted learning was associated with higher video telehealth use, programs offering in-person technical support for initial setup, device maintenance, and follow-up training may be critical. While family and peer support systems have shown promise (Kuoppamäki et al., 2022; Wilson et al., 2021), this may not be feasible for homebound older adults as they often experience social isolation (Ankuda et al., 2022; Cudjoe et al., 2022). In such cases, formal support systems become critical, and in-home training services could be highly beneficial, considering the limited mobility of homebound older adults. A notable implementation of this approach is *Talking Tech*, a collaborative initiative between a program development company, an NGO, and a university research institute (Gadbois et al., 2022). Talking Tech combines regular meal deliveries to homebound older adults with volunteer-led training on operating ICT devices and accessing online programs, effectively enhancing digital literacy and reducing social isolation.

This study offers valuable insights but also leaves room for further exploration. First, while our analysis focused on eHealth use during the COVID-19 pandemic, future studies should examine post-pandemic trends to better understand the long-term evolution of eHealth adoption. Second, we measured usage only as binary engagement (yes/no), without capturing frequency or intensity of use—important factors for understanding dependency and digital routines. Third, although we categorized users by modality, we did not examine how each type of eHealth use relates to health outcomes. Future studies should assess whether patient portal or telehealth use leads to better outcomes, and how these relationships differ by sociodemographic characteristics or degrees of homebound status (e.g., “rarely” vs. “never” leaving home). Fourth, while we treated ICT access, prior online experience, and ICT training as distinct variables, these factors likely interact. Individuals with experience or training likely have device access, suggesting that the observed effects may partially reflect overlapping pathways. Future research should explore whether the sequence of access (e.g., gaining skills before acquiring a device vs. vice versa) shapes engagement differently.

Despite these limitations, this study contributes important insights by distinguishing between types of eHealth use and examining ICT-related predictors among homebound older adults during the pandemic. While previous research has examined digital health among older adults and those with functional limitations, this is the first study focusing on the homebound population. The findings have implications that extend beyond COVID-19. Indeed, even before the pandemic, eHealth was identified as a promising tool for reaching those unable to attend in-person visits. Researchers and policymakers must continue addressing eHealth disparities in this underserved population to ensure equitable access to healthcare in the digital age.
